# Suggested Methods of Development

**Published:** 1921-08-20

**Authors:** 


### "MASS" CONTRIBUTIONS IN LONDON.

### Suggested Methods of Development.

King Edward's Hospital Fund for London has issued a circular dated August 11 to London Hospitals on (1) Collections from Employees and (2) Co-ordination of Appeals, as distinct from organised collections from employees. In regard to (2) the King's Fund is not as yet prepared to make any general statement. It is conferring with the other central funds and agencies, and a standing Advisory Committee thereof is to be formed, to which questions arising in this connection may be referred. The hospitals are to be represented on this Committee through the London Regional Committee of the British Hospitals Association. The King's Fund recognises that it will take time to work out principles on which the co-ordination of appeals can be put into effective practice without affecting the efforts of the central funds and separate hospitals, and it states that meantime the individual hospitals are free to act on their own judgment in the matter of appeals other than for capital expenditure. It would, however, be convenient if hospitals which are contemplating special appeals were to notify the King's Fund, which would then be in a position, should occasion arise, to give information which might help to prevent overlapping.

### Collections from Employees.

Following on the resolution passed at the Conference of hospital representatives at the Mansion House on July 6 [The Hospital, July 16, page 264], the King's Fund Policy Committee has held conferences with representatives of the central agencies—*i.e.*, the London Regional Committee of the British Hospitals Association, the Hospital Sunday Fund, the Hospital Saturday Fund, and the League of Mercy. It points out that one starting-point of the whole discussion is the opinion of Lord Cave's Committee that a larger development of collections from wage-earners is possible in the London area; another is the opinion expressed by the Conference of hospital representatives just referred to that the method which should be tried is that of co-operation between the hospitals and the central agencies in local collections from employees. It may be that the development of collections will come either from an intensification of the present efforts of the hospitals and the central agencies or from a change in their methods. These are all points for discussion, and the King's Fund Policy Committee and the Conference of Central Agencies take the view that it will be best not to pass binding resolutions on any point until more evidence on many important aspects of the subject is obtained. The discussion of practical details should provide answers to such questions as how far combined action is necessary and what is the best method of obtaining it, and should produce more evidence as to the forms of organisation and contribution likely to be acceptable both to hospitals and contributors, as well as workable and likely to produce the desired result. The existence of provident schemes alongside non-provident schemes is another matter on which more light is needed.

The Organisation of Hospital Areas.

In the conferences which are proceeding at present between the central agencies certain suggestions have been made, and discussions are proceeding experimentally on their basis. They are (1) that for the purposes of collections from employees the Metropolis (nine miles of Charing Cross) should be divided into hospital areas, ranging round key hospitals, and that all or some of the key hospitals might co-operate with the Hospital Saturday Fund and the League of Mercy in the organisation of collections from employees in their areas; (2) in each hospital area with a key hospital a committee should be formed in connection with the hospital or hospitals consisting of the local representatives of the Hospital Saturday Fund, the League of Mercy, and other organisations, whose function should be to co-operate in securing the maximum collections from the employees in the area; (3) hospitals actively co-operating with these two agencies should be assured of a due proportion of the proceeds of the local collections from employees. The Conference is still discussing the question whether any change is required in the practice as regards weekly contributions from employees in view of the increased cost of hospital treatment and the development of contributions from patients. What was an adequate contribution by an employee in 1914 towards the provision of free treatment when needed cannot now, in view of the changed circumstances, be considered adequate. Should the remedy be sought in increased weekly contributions, or in weekly contributions combined with patients' payments? This is under discussion.

Local Conferences to be Called.

To assist in the consideration of the question of organisation by areas the King's Fund has issued a list of London hospitals, geographically arranged, with the adjacent municipal areas and the Hospital Saturday Fund and League of Mercy areas. This table is not intended to convey suggestions as to possible organisation areas, but the King's Fund suggests that in the discussion of the above suggestions by the hospitals, either singly or by means of conferences between neighbouring hospitals and local agencies, including representatives of the Hospital Saturday Fund and the League of Mercy, and the clergy and ministers who support the Hospital Sunday Fund, it would be convenient if all hospitals would make use of it; this would help in maintaining the relation between local movements and the general movement. Some hospitals already contemplate the holding of local conferences, and the King's Fund Policy Committee offers to assist in the convening of others.

The King's Fund Policy Committee will welcome any observations which the committee of any hospital may desire to make either as an expression of the individual opinion of the hospital or as the result of discussion with other hospitals or other agencies. Meanwhile it asks for information on four specific points: (1) What steps each hospital has taken in the past to obtain systematic contributions from employees; (2) within what area and (3) with what results; (4) what special experience has been gained of the difficulties and of methods of overcoming them.

